# LA structural remodeling is predicted by arterial stiffening independently of conventional risk factors

**DOI:** 10.1186/1532-429X-18-S1-Q36

**Published:** 2016-01-27

**Authors:** Miguel S Vieira, Bram Ruijsink, Isma Rafiq, Marina Cecelja, C Alberto Figueroa, Tarique Hussain

**Affiliations:** 1 grid.13097.3c0000000123226764Division of Imaging Sciences & Biomedical Engineering, King's College London British Heart Foundation Centre, London, United Kingdom; 2 grid.214458.e0000000086837370Departments of Surgery and Biomedical Engineering, University of Michigan, Ann Arbor, MI USA; 3 grid.13097.3c0000000123226764Department of Clinical Pharmacology, King's College London British Heart Foundation Centre, London, United Kingdom; 4 Pediatric Cardiology, University of Texas, Dallas, TX USA

## Background

Left atrium (LA) size and function are powerful biomarkers of cardiovascular outcomes in many diseases. We sought to determine if the expected age-associated increase in arterial stiffness (AS) and left ventricular (LV)-LA afterload leads to corresponding effects on LA function and this can be measured with cardiovascular magnetic resonance (CMR). Additionally, we investigated the significance of these markers in asymptomatic individuals with cardiovascular risk factors (CRF).

## Methods

Female subjects from the Twins UK cohort with no overt cardiac disease were prospectively recruited for a CMR study on a 1.5 Tesla scanner (Philips, Best, Netherlands) with tissue characterization (T1 mapping and late gadolinium enhancement). Patients with atrial fibrillation, valvular disease, regional wall motion abnormalities at rest or areas of myocardial enhancement were excluded from the analysis. LA reservoir, conduit and contractile functions were quantified by both fractional volume changes and CMR feature tracking derived strain and strain rate. Additionally, CMR feature tracking derived myocardial deformation indices and pulse wave velocity (PWV) (foot-to-foot methodology), were calculated.

## Results

**Figure 1:**
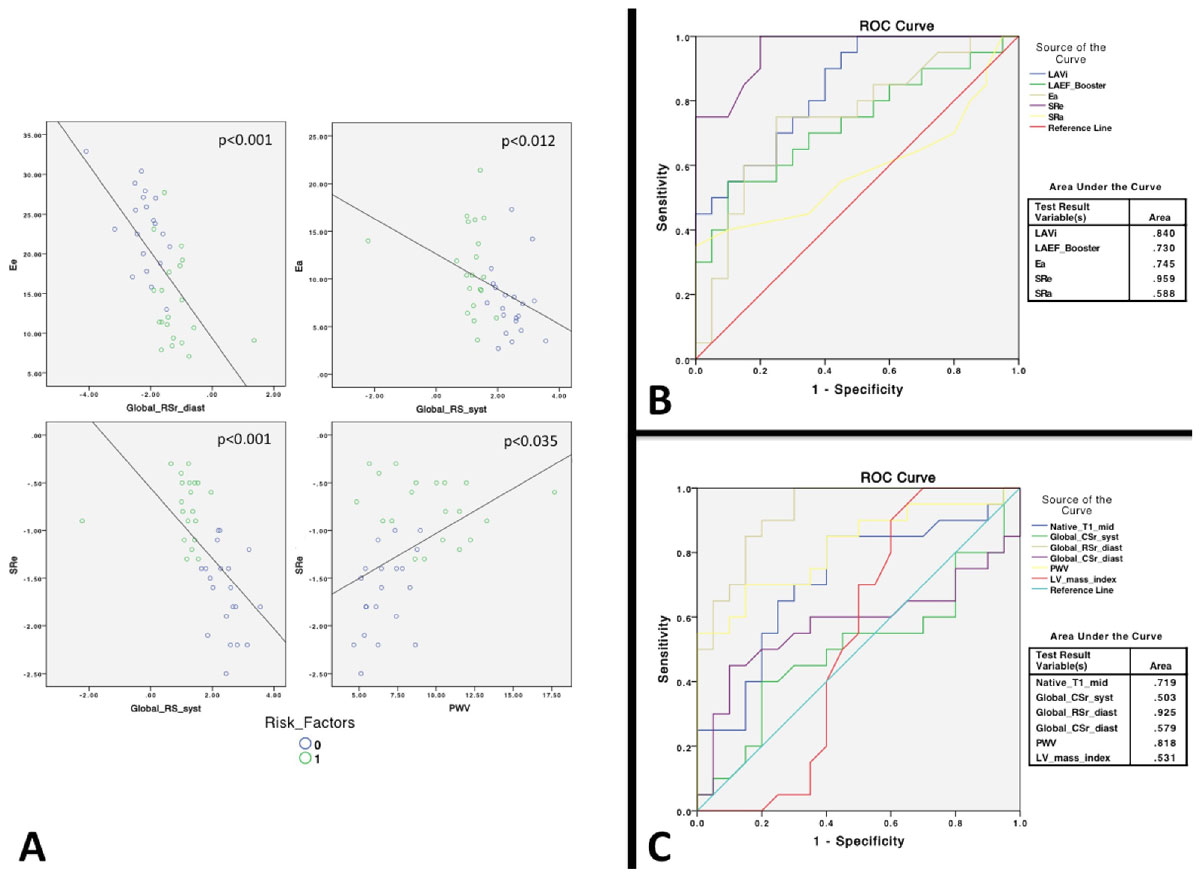
**Scatter plots (Panel A) showing the relationships between LA-LV deformation parameters and LA-PWV (abbreviations as shown in table** 1**)**. GRs (syst), GRSr (diast), and PWV were identified as independent determinants of LA mechanics in individuals with risk factors on multivariate analysis. **Panels B and C.** ROC curves comparing the sensitivity and specificity of different diagnostic parameters used in the analysis in the presence of cardiovascular risk factors.

40 female twins were enrolled. Baseline characteristics are shown in table 1. Bivariate analysis showed that LA volume, LA reservoir, conduit and booster function correlated with LV deformation parameters and with PWV, a surrogate marker of AS (p < 0.001 to 0.044). Furthermore, LA function components assessed by fractional volume changes were significantly different in the presence of ≥1 risk factors (p < 0.001 to 0.012). Multivariable regression analysis confirmed that only the conduit and booster components were associated with changes in LV deformation and PWV (figure 1, panel A). Subjects with CRF had lower LA conduit function and higher booster function. We hypothesize that this can be attributed to an increase in atrial afterload in response to increased LV stiffness-AS that occurs with aging but is more pronounced subjects with CRF as is demonstrated by lower LV deformation indices and higher calculated PWV. ROC analysis showed that LA volume and function parameters outperformed LV deformation and AS parameters in the evaluation of subclinical cardiac changes in the presence of CRF (figure 1, panels B and C)

**Table 1:** Baseline characteristics

CHARACTERISTICS	ALL	CRF = 0	CRF≥1	p
Age, years	69 ± 5.6 [57-77]	68 ± 5.6	69 ± 5.6	0.114
Women, n (%)	40 (100%)	20	20	-
Cardiovascular risk factors (≥1)	20 (50%)	0	20	<0.001
Hypertension, n (%)	9 (21%)	0	9	0.0012
Diabetes, n (%)	0	0	0	-
Dyslipidemia	16 (40%)	0	16	<0.001
Smoker (former), n (%)	4 (10%)	0	4	0.106
Pulse wave velocity [PWV (m/s)]	8.127 ± 2.77	6.630 ± 2.77	8.127 ± 3.03	<0.001
LV end-diastolic volume indexed [LVEDVi (mL/m2)]	70 ± 14.5	67 ± 8.4	71 ± 13.1	0.079
LV end-systolic volume [LVESVi (mL/m2)]	41 ± 9.6	41 ± 6.4	35 ± 9.6	0.153
LV ejection fraction [LVEF (%)]	67 ± 7.4	61 ± 7.4	70 ± 7.4	0.975
LV mass indexed (mg/m2)	54 ± 12.5	53 ± 12.5	55 ± 6.4	0.637
Global circumferential strain (GCs)	-20.5 ± 8.6	-24.7 ± 10.4	-16.4 ± 3.5	0.001
Global radial strain (GRs)	29.8 ± 12.0	30.7 ± 6.9	28.9 ± 15.7	0.652
Global circumferential strain rate peak systole [GCSr (syst)]	-1.58 ± 0.42	-1.58 ± 0.41	-1.57 ± 0.45	0.924
Global radial strain rate peak systole [GRSr (syst)]	1.77 ± 0.96	2.44 ± 0.50	1.09 ± 0.83	<0.001
Global circumferential strain rate early diastole [GCSr (diast)]	2.17 ± 1.90	1.85 ± 0.52	2.47 ± 2.6	0.293
Global radial strain rate early diastole [GRSr (diast)]	-1.7 ± 0.83	-2.21 ± 0.61	-1.2 ± 0.71	<0.001
Native T1 (mid septum, ms)	979 ± 37.6	966 ± 30.6	993 ± 39.9	0.022
LA volume indexed [LAVI (mL/m2)]	42 ± 14.0	34 ± 9.6	50 ± 13.4	<0.001
LA ejection fraction total [LAEF total (%)]	61 ± 11.6	68 ± 7.8	53 ± 9.9	<0.001
LA conduit function (%)	34 ± 12.0	42 ± 10.6	26 ± 7.7	<0.001
LA booster pump function (%)	33.3 ± 9.0	30 ± 6.8	37 ± 9.7	0.012
LA total strain (εs)	28.0 ± 8.0	30.7 ± 7.4	25.2 ± 7.9	0.029
LA passive strain (εe)	18.6 ± 7.2	23.3 ± 5.2	14.0 ± 5.7	<0.001
LA active strain (εa)	LA active strain (εa)	7.5 ± 3.6	11.2 ± 4.6	0.006
LA peak positive strain rate (SRs)	1.2 ± 0.4	1.4 ± 0.4	1.1 ± 0.3	0.016
LA peak early negative strain rate (SRe)	-1.2 ± 0.6	-1.7 ± 0.4	-0.8 ± 0.3	<0.001
LA peak late negative strain rate (SRa)	-1.0 ± 0.4	-1.0 ± 0.3	-0.9 ± 0.4	0.341

CRF, cardiovascular risk factors.

## Conclusions

LA mechanics correlates with LV deformation parameters and PWV and differs significantly in elderly subjects with CRF compared to their healthy age-matched peers. LA structural remodeling is predicted by AS (as expressed by PWV/LV parameters) independently of conventional CRF, thus supporting the hypothesis of arterial-ventricular-atrial coupling (AVAC). These novel markers of LA performance can potentially uncover abnormal AVAC in patients with CRF but no overt cardiac disease and give valuable insights into ventricular dysfunction beyond standard volumetrics.

